# 91. Knowledge, Attitudes, and Practice of Antibiotic Prescribing among Nurse Practitioners

**DOI:** 10.1093/ofid/ofab466.293

**Published:** 2021-12-04

**Authors:** Siobhán Brennan, Elizabeth Walters, Sydney E Browder, Ravi Jhaveri, Zach Willis

**Affiliations:** 1 University of North Carolina Chapel Hill, Durham, NC; 2 University of North Carolina School of Nursing, Chapel Hill, NC; 3 UNC Gillings School of Public Health, Durham, NC; 4 Northwestern University Feinberg School of Medicine; Ann & Robert H. Lurie Children’s Hospital of Chicago, Chicago, IL; 5 University of North Carolina, Chapel Hill, NC

## Abstract

**Background:**

Antibiotic overuse (AO) in ambulatory care is an important public health problem. Nurse practitioners (NPs) account for a growing proportion of outpatient antibiotic prescriptions: 14.6% in 2016. Our objective was to assess NPs’ attitudes about antibiotic prescribing practices and knowledge and use of antibiotic prescribing guidelines (APG) in their practice.

**Methods:**

We distributed a survey via email to NPs listed as licensed by the North Carolina Board of Nursing. Surveys were distributed three times; duplicate responses were not permitted. Respondents who reported not prescribing antibiotics in the outpatient setting were ineligible. Three randomly selected respondents received gift cards. Questions assessed degree type, practice type, years in practice, and attitudes about antibiotic prescribing practices antibiotic stewardship. Respondents answered four questions assessing knowledge of APG. Analyses were descriptive; scores on knowledge questions were compared using T-tests.

**Results:**

Survey requests were sent to 10,094 listed NPs; there were 846 completed responses (8.4%), of which 672 respondents (79.4%) reported prescribing antibiotics in outpatient care. Of those, 595 (88.5%) treat adult patients. Most respondents agreed that AO is a problem in their state (84.5%); 41.3% agreed that it was a problem in their practice. Patient/family satisfaction was the most frequently reported driver of AO (90.1%). Most respondents agreed that national APG are appropriate (95.4%) and that quality improvement (QI) is warranted (93.4%). Respondents reported following APG always (18.5%) or more than half the time (61.0%). Respondents answered a mean of 1.89 out of 4 knowledge questions correctly, with higher scores among those reporting following APG more than half the time (1.97 vs 1.58, p< 0.0001).

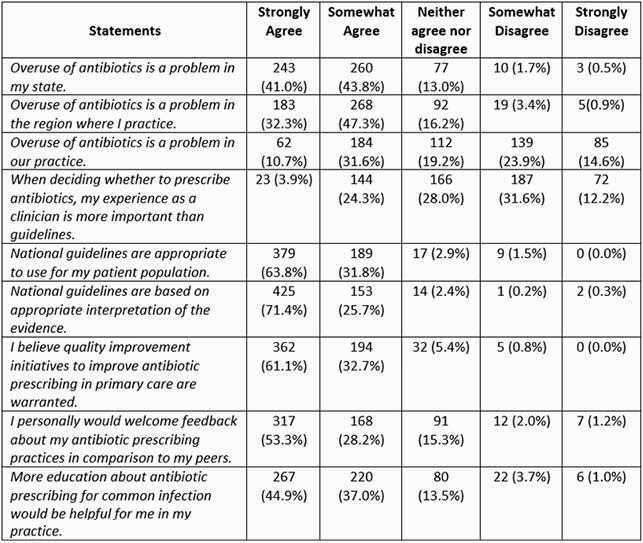

Overall attitudes about antibiotic prescribing, antibiotic prescribing guidelines, and acceptance of Quality Improvement. N=595.

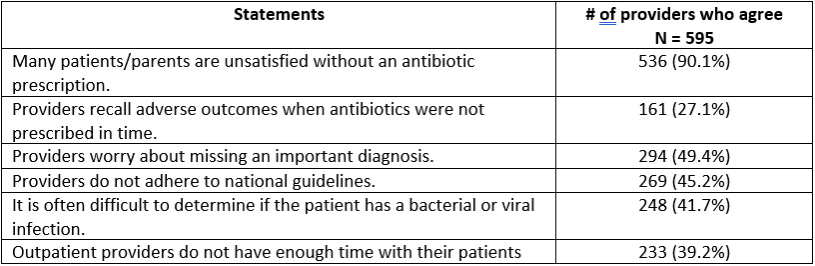

Respondents’ reported drivers of antibiotic overuse. Respondents were permitted to select more than one driver.

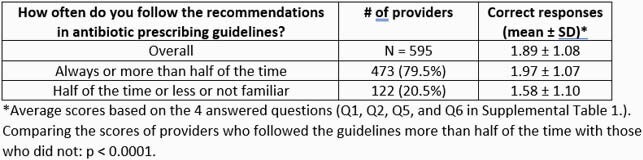

Content question performance by self-reported guideline compliance; scores represent the number correct out of four questions.

**Conclusion:**

Respondents agree that AO is a problem but place responsibility externally. Confidence in APG was high; most respondents endorsed following APG most of the time. Performance on knowledge questions suggests a need for education. Most respondents would welcome QI focused on AO, including education and personalized feedback. Similar work is needed in other regions and among other prescriber groups. The results will inform outpatient antibiotic stewardship.

**Disclosures:**

**Elizabeth Walters, DNP, CPNP-PC, RN**, **Merck** (Consultant, Other Financial or Material Support, I am a trainer for the Nexplanon product.) **Ravi Jhaveri, MD**, **AstraZeneca** (Consultant)**Dynavax** (Consultant)**Elsevier** (Other Financial or Material Support, Editorial Stipend as Co-editor in Chief, Clinical Therapeutics)**Seqirus** (Consultant)

